# Gut Microbiome, Neuroinflammation, and Fetal Alcohol Spectrum Disorders: Insights from Rodent Models

**DOI:** 10.3390/biology14060593

**Published:** 2025-05-23

**Authors:** Abdulrahman M. Busayli, Wenhua Xu, Ghaidaa A. Raffah, Gang Chen

**Affiliations:** 1 Department Pharmacology & Nutritional Sciences, University of Kentucky College of Medicine, Lexington, KY 40536, USA; 2 Department of Clinical Nutrition, College of Nursing and Health Sciences, Jazan University, Jazan 82817, Saudi Arabia

**Keywords:** ethanol, fetal alcohol spectrum disorders, gut microbiome, neuroinflammation, short-chain fatty acids, brain, rodent, behavior

## Abstract

Fetal alcohol spectrum disorder (FASD) is a significant public health issue that affects children. It results from ethanol exposure during pregnancy, leading to considerable physical, neurodevelopmental, behavioral, and cognitive deficits. The exact mechanism is not well understood. Recently, considerable attention has been focused on the influence of gut microbiome on brain development through the gut–brain axis. Changes in the gut microbiome resulting from ethanol exposure may contribute to the pathology of FASD, potentially involving neuroinflammation. This literature review summarizes the existing research and primary animal studies on the impact of early ethanol exposure on the gut microbiome, neuroinflammation, brain development, and behavioral consequences. The evidence suggests that early ethanol exposure alters the gut microbiome, which may induce neuroinflammation, brain damage, and cognitive impairment. However, a clear causal relationship among these factors remains to be fully elucidated.

## Simple Summary

Fetal alcohol spectrum disorder (FASD) is a serious condition that affects children whose mothers drank alcohol during pregnancy. It can lead to problems with physical growth, brain development, behavior, and learning. Studies are now exploring how alcohol might harm the baby by first disrupting the mother’s gut bacteria, which may then affect the baby’s brain through what’s known as the gut–brain connection. This review focuses on studies in animals to better understand how early alcohol exposure might lead to brain inflammation and long-term problems with thinking and behavior. While there is growing evidence of these links, more research is needed to clearly understand how they are connected.

## 1. Introduction

FASD is a range of disorders that result from maternal alcohol consumption during pregnancy. These disorders can influence a child’s physical, behavioral, and cognitive development [1]. The incidence of children with FASD is notably significant, with an estimation that 8 out of every 1000 children are affected by FASD [2]. Children with FASD frequently encounter numerous health issues, such as cognitive impairments, memory problems, and behavioral disorders. These health issues place significant stress on families as they manage the challenges associated with these children, including sleep disturbances, eating disorders, toileting difficulties, and speech and language impairments [3]. The entire society, including the healthcare system, may be impacted, as the care of individuals with FASD represents a financial burden. The estimated yearly costs for caring for individuals with FASD in the USA range from USD 926 million to USD 3.2 billion [4]. Ongoing efforts have been undertaken to fully understand FASD but the specific mechanisms involved in its development remain unclear, underscoring the need for further investigation.

The research on gut microbiota is potentially significant due to its vital function in brain development through the gut–brain axis. The gut microbiome, consisting of various microorganisms in the digestive system, plays a crucial role in maintaining overall health and well-being. These microbes regulate immune responses and synthesize essential metabolites, including vitamins, neurotransmitters, and short-chain fatty acids (SCFAs), which significantly influence the gut and other distant organs, such as the lung, liver, and brain [5]. Research has provided evidence demonstrating that an imbalance in gut microbiome, including changes in the composition and function of the gut microbiome, might be linked to neurological and developmental conditions, such as attention deficit hyperactivity disorder (ADHD) [6] and autism spectrum disorder (ASD) [7]. According to Knag et al., there were apparent changes in the gut microbiome in children with ASD, as they experienced a reduction in the levels of beneficial bacteria and an increase in the levels of potentially harmful bacterial families [8]. These neurological and developmental conditions (ASD and ADHD) interestingly share key features with FASD, such as social communication challenges, cognitive impairment, and difficulties with executive functioning [1,9]. Studying the gut–brain axis in FASD may help to understand its etiological mechanisms and identify potential treatment strategies.

The gut–brain axis is a bidirectional communication network that connects the gastrointestinal tract to the central nervous system. It helps regulate the brain’s development and function through mechanisms including metabolic, endocrine, neural, and immune pathways [10]. Microbes produce metabolites, such as neurotransmitter precursors and SCFAs, which play a significant role in this communication network [11]. These metabolites play a crucial role in brain development by regulating various biological processes, which in turn influence behavioral responses and cognitive function [12]. The gut–brain axis is a vital structure for understanding multiple neurodevelopmental disorders. According to Wang et al. (2023), alterations in gut–brain axis communication may profoundly influence the brain function [13]. Therefore, understanding this complex network function is crucial, as it can provide important insights into neurological and neurodevelopmental disorders, particularly in the context of FASD, a condition resulting from alcohol exposure that affects multiple biological systems.

Neuroinflammation resulting from maternal ethanol exposure during pregnancy is regarded as a primary mechanism involved in the neurodevelopmental effects observed in FASD. This neuroinflammation contributes to structural and functional brain abnormalities, resulting in cognitive and behavioral deficits [14]. Although the direct effect of ethanol on neuroinflammation is well established, the impact of the gut microbial network remains unknown, highlighting an area that requires further research. A comprehensive understanding of the complex relationships between neuroinflammation and the gut–brain axis could provide new approaches for addressing conditions associated with FASD and underscore potential treatment alternatives.

Several potential benefits of using rodent models in FASD research are noted. One of the main reasons is that these models have a strong resemblance to human brain development and have the ability to replicate alterations in the gut microbiome [15,16]. In addition, these models, upon exposure to ethanol, exhibit similar health conditions to those observed in FASD, including neuroinflammation, behavioral changes, and cognitive impairment [17,18,19,20,21]. Furthermore, these models enhance our understanding of the connection between gut microbiome imbalances and neurodevelopmental outcomes, as they allow for precise control in research experiments. Lastly, various potential intervention approaches, such as SCFA supplementation and probiotics, have been identified through research using these models [22,23].

This review analyzes evidence from rodent models to explore the correlation between gut microbiome dysbiosis and neuroinflammation in FASD. It explores the effect of maternal ethanol consumption on the gut–brain axis and its role in FASD. Our goal is to enhance the understanding of these systems, identify potential interventions, and bridge the existing gaps.

## 2. Gut Microbiome and FASD

### 2.1. The Role of Gut Microbiota in Brain Development and Function

The human gastrointestinal tract is a highly complex organ system that includes the intestinal epithelium, microorganisms, and the mucosal immune system [24]. The gut microbiome is a general term that describes the diverse community of microorganisms residing in the gastrointestinal tract, comprising bacteria, fungi, archaea, and viruses. Bacteria are regarded as the most abundant component, accounting for up to 99% of the total gut microbial population [25]. These microbial communities play a primary role in human health by converting nutrients into metabolites, which can have positive and negative effects. The metabolites produced by these microbes interact with the host, affecting health status and disease progression [26]. In addition to supporting gastrointestinal health, the gut microbiome affects other organs beyond the gastrointestinal tract. One such organ is the brain, which receives signals through gut microbiome metabolites, underscoring the systemic influence of the gut microbiome on the body [27].

The gut microbiome significantly influences brain function in various ways. This impact is associated with the gut–brain axis, a vital system that facilitates the production and regulation of neurotransmitters [27]. The gastrointestinal tract and brain are connected through the bidirectional communication of the gut–brain axis, enabling a reciprocal relationship. The gut microbiome can send signals that impact the brain’s function and behavior. Similarly, the brain sends signals that can affect the composition and function of gut microbes [28]. In addition, the gut microbiome plays a significant role in the function and development of the immune system through various pathways and metabolites that support brain homeostasis and influence overall brain function [29].

Studies using germ-free (GF) mice have shown that brain function is significantly affected by the absence of a gut microbiome in these mice [30,31]. GF mice demonstrated increased motor activity and reduced anxiety-like behavior [30]. However, after 24 h of exposure to the outside environment, these GF mice became less anxious, while their locomotor activity remained unchanged [31]. Furthermore, these GF mice exhibited alterations in gene expression pathways, primarily those associated with second messengers and synaptic long-term potentiation, which are crucial for controlling motor and anxiety behaviors [30]. Additionally, a reduction in neurotransmitters, particularly dopamine, serotonin, and norepinephrine, was observed in GF mice in most brain regions [31]. These findings collectively emphasize the significance of the relationship between the gut microbiome and the brain, as evidenced in GF mice that lack a gut microbiome, and how this significantly impacts brain function.

The connection between brain development and the gut microbiome is particularly crucial during early life, as shown by the developmental similarities between the brain and the gut [32]. The gut microbiome demonstrates high diversity and richness during the early developmental stages. Disruption during this critical period can significantly impact the brain’s function and development. Heijt et al. (2011) demonstrated that introducing the gut microbiome of GF mice early in life normalized their behavioral and molecular changes, making them similar to specific pathogen-free mice that have a normal gut microbiome [30]. In contrast, introducing the gut microbiota to adult GF mice did not restore behavioral and neurological abnormalities. These findings suggest that proper colonization of the gut microbiome must occur during early developmental stages, since it significantly influences brain development and behavioral regulation during this period.

The specific composition of the gut microbiome, not just its presence, has a significant influence on cognitive development. High levels of *Bacteroides* have been linked to improved cognitive outcomes in children. In contrast, individuals with high alpha diversity in the gut microbiome tend to have lower scores on overall composite measures, expressive language scales, and visual reception scales [33]. Although the gastrointestinal microbiome is crucial during early development, some bacteria may have a more advantageous impact than others. For example, during the early developmental stage, beneficial bacteria such as *Bifidobacterium* are essential because they help regulate the synaptic expression of genes and microglial activity in many vital brain regions [34]. In contrast, the overgrowth of specific bacteria, such as *Proteobacteria*, negatively affects the development process, which is often associated with an inflammatory state that can influence early neurodevelopmental processes [35]. These results suggest that the diversity and balance of the gut microbiome are essential, as they may contribute to achieving favorable cognitive outcomes.

Many additional factors, such as maternal stress, environmental chemicals, medications, and diet, profoundly affect gut microbiome composition and diversity during the early developmental period [36]. Leclercq et al. (2017) reported that administering low doses of antibiotics to mice during late pregnancy and the early postnatal period resulted in long-term alterations in neurochemistry and behavior [37]. These antibiotics caused lasting alterations in the gut microbiome, as well as effects on the integrity of the blood–brain barrier and cytokine expression in the brain. These treated mice also exhibited impaired anxiety and social behavior, in addition to a tendency to display aggression. Furthermore, another study found that significant anxiety-related stress and behavioral changes in neonatal mice subjected to maternal separation led to alterations in gut microbiome composition [38]. These findings suggest that the early life environment significantly influences the composition of the gut microbiome, which could have lasting effects on brain function and behavioral consequences.

### 2.2. Short-Chain Fatty Acids

The gut microbiome affects brain health via intricate mechanisms, with SCFAs as key mediators. The anaerobic fermentation of dietary fibers by intestinal bacteria generates SCFAs, primarily acetate, butyrate, and propionate [39]. In the human gastrointestinal tract, the colon holds the highest concentration of SCFAs, with typical proportions of about 20% propionate, 60% acetate, and 20% butyrate [40]. The specific levels vary among people depending on microbiome composition and dietary fiber content, with concentrations reaching up to 50–200 mmol/L [39].

SCFAs can affect the central nervous system due to their ability to pass the blood–brain barrier (BBB) through monocarboxylate transporters, which are abundantly found in endothelial cells [29]. They play crucial roles in the central nervous system by helping maintain neural homeostasis and regulating immune system responses. Two primary mechanisms regulate their function: binding to G protein-coupled receptors and inhibiting histone deacetylases [41,42]. For instance, SCFAs, particularly butyrate, have been observed to lower neuroinflammation by inhibiting histone deacetylase in a mouse model of ischemic stroke and BV2 microglial cells [43]. The research indicated that butyrate transitioned microglia from a pro-inflammatory to an anti-inflammatory state via altering histone acetylation (H3K9ac) patterns at critical gene promoters. It decreased acetylation at the TNF-α and STAT1 genes, which contributed to the upregulation of the anti-inflammatory IL10/STAT3 signaling pathway and the downregulation of pro-inflammatory mediators (TNF-α, NOS2), ultimately leading to significant neuroprotection in the ischemic brain.

SCFAs also play an important role in brain development. Butyrate has been shown to promote neurogenesis by increasing the synthesis of brain-derived neurotrophic factors, hence supporting synaptic plasticity and neuronal health survival [44]. Furthermore, propionate plays a role in maintaining the integrity of the BBB, providing essential protection for the developing brain [45]. Additionally, acetate regulates microglial cells by diminishing inflammatory signaling via the reduction in pro-inflammatory cytokines and the inhibition of inflammatory signaling pathways [45]. A recent study underscores the essential function of SCFAs in brain development, revealing that antibiotic-induced gut dysbiosis in neonatal mice led to diminished fecal SCFA levels. These mice showed impaired cognitive function, which improved when the balance of the gut microbiome was restored [46]. These findings underscore the importance of maintaining appropriate SCFA levels through gut microbiome health for optimal brain development.

### 2.3. Effects of Ethanol Exposure on Gut Microbiome

Studies on alcohol consumption in adults have established clear patterns of gut dysbiosis. A recent cohort study analyzing data from 4575 adults, both male and female (aged 25–74), found that high alcohol consumption was associated with gut dysbiosis, marked by a decrease in beneficial bacteria, increased Gram-negative (potentially harmful) bacteria, and changes in overall the microbiome composition that suggest compromised intestinal barrier function and increased inflammation [47]. In pregnant individuals, alcohol exposure produces similar taxonomic shifts but with additional consequences, as maternal alcohol consumption significantly alters both maternal and infant gut microbiota composition. A study demonstrated that mothers who consumed alcohol during pregnancy showed a decreased abundance of beneficial bacteria, which have anti-inflammatory properties, and increased levels of other bacterial taxa, which are associated with intestinal permeability and hepatic diseases [48]. Unlike the reversible dysbiosis seen in non-pregnant adults once alcohol consumption stops, the consumption of alcohol in pregnant adults induces maternal microbiome alterations that can lead to lasting dysbiosis in offspring, as demonstrated in the following research.

Recent research utilizing Sprague Dawley rat models has revealed significant evidence of lasting alterations in the gut microbiome subsequent to ethanol exposure during pregnancy. The gut microbiome compositions of offspring were analyzed at postnatal day 80 via 16s rRNA [49]. This study revealed a significant and sex-specific alteration in the gut microbiome. While males showed changes in *Bacteroides* and *Bifidobacterium* populations, females showed an alteration in *Faecalitalea* and *Proteus* [49]. Regarding these findings, research on sex-based differences in FASD microbiome patterns remains limited. This presents a critical knowledge gap, especially considering evidence from other conditions that showed that sex hormones significantly influence microbial communities [50], with males showing greater microbiome sensitivity to environmental challenges, as evidenced in models of inflammation and autoimmunity [51,52]. These sex differences in microbial composition found in the offspring indicate that maternal ethanol exposure may influence the development of the gut microbiome in a sex-dependent manner, potentially leading to sex-specific vulnerabilities in neurodevelopmental outcomes. The timing of these changes is crucial, as maternal exposure to ethanol influences the establishment of the gut microbiome in early life, resulting in alterations that have long-lasting effects continuing into adulthood rather than resolving. This suggests that maternal ethanol exposure induces lasting changes in the offspring’s gut microbiome, which may continuously disrupt gut–brain communication and contribute to the development of FASD.

The impact of maternal ethanol exposure on gut microbiome extends beyond simple compositional changes to affect key metabolic functions. Subsequent analyses of the samples collected on gestational day 14 and postnatal day 22 (P22) demonstrated that ethanol-exposed dams showed a decreased abundance of important butyrate-producing bacterial families, including *Ruminococcaceae* and *Lachnospiraceae* [53]. Conversely, neonatal rats exposed to ethanol exhibited a greater prevalence of *Desulfovibrionaceae*, a bacterial family that produces SCFAs like propionic acid, mainly through the genera *Clostridia*, *Bacteroidetes*, and *Desulfovibrio*, which have been linked to exacerbated ASD-related behaviors [54]. Furthermore, both FASD and ASD exhibit a decreased presence of beneficial bacteria, such as *Prevotella*, indicating shared pathways of microbial disruption [53,55]. These findings suggest that maternal ethanol exposure may play a crucial role in the pathophysiology of FASD due to gut dysbiosis, which impacts bacterial composition and metabolic pathways, indicating potential similarities in mechanisms with ASD.

## 3. Neuroinflammation in FASD

### 3.1. Ethanol-Induced Neuroinflammation

Although several factors contribute to the etiology of FASD, neuroinflammation is regarded as a primary factor in the development of FASD. It is defined as the brain’s response to various stimuli, characterized by the activation of immune cells—primarily astrocytes and microglia—and the production of pro-inflammatory molecules [56]. In cases of FASD, exposure to alcohol during pregnancy induces a complex cascade of inflammatory responses in the developing brain, creating a lasting inflammatory state due to the sustained activation of immune cells and excessive production of pro-inflammatory molecules [57]. This neuroinflammation may have long-term effects that extend beyond the initial exposure, leading to alterations in brain structure and function that manifest as the cognitive and behavioral deficits observed in FASD [14].

### 3.2. Cellular Mediators of Ethanol-Induced Neuroinflammation

Exposure to ethanol during pregnancy may activate two primary immune cells, microglia and astrocytes, in the central nervous system (CNS), resulting in the production of pro-inflammatory cytokines by these cells. Microglia are regarded as the primary immune cells in the CNS, which undergo intricate changes in their function and morphology due to ethanol exposure during development [56]. In a normal surveillance state, microglia have small cell bodies with long, extensive branching processes that continuously monitor the brain environment [56]. However, exposure to ethanol causes specific temporal and regional changes in the microglia through several mechanisms. Morphologically, ethanol prompts these cells to transition from their resting state to an activated phenotype, characterized by shortened processes, hypertrophic somas, and broader cell bodies [56,58]. These changes have been observed in the cerebellum, hippocampus, and cerebral cortex [59]. Ethanol at the molecular level promotes the TLR4/TLR2 association in microglia, inducing inflammatory responses by increasing the production of pro-inflammatory molecules such as IL-1β, TNF-α, and several chemokines [60,61]. Microglia activation may disrupt their normal developmental functions, such as synaptic pruning and surveillance [62], significantly affecting crucial neurodevelopment processes necessary for proper brain function.

Ethanol exposure may also affect astrocytes, which play a crucial role in immunoregulation and contribute to the neuroinflammatory processes associated with FASD pathophysiology [58]. Ethanol exposure may trigger reactive astrogliosis in astrocytes, characterized by cellular hypertrophy and an increase in the production of glial fibrillary acidic protein (GFAP) [63,64]. Astrocyte activation has been observed in specific brain areas, particularly the hippocampus and cortex, in a study that involved administering ethanol to newborn mice via vapor inhalation for 4 h on postnatal days 3 to 5, resulting in a blood ethanol concentration of 0.5 g/dL [65]. Additionally, the activation of astrocytes may increase the production of several pro-inflammatory chemicals, including IL-1β, cyclooxygenase 2, and iNOS. This induces modified inflammatory signaling pathways through the activation of transcription factors AP-1, NF-κB, IL-1R-associated kinase, ERK1/2, p38, and JNK kinases [66,67,68]. Furthermore, the activation of astrocytes may disrupt calcium signaling in both neurons and astrocytes, resulting in neurotransmitter imbalance and contributing to FASD [57].

### 3.3. Impact of Neuroinflammation on Brain Development

Neuroinflammation during the developmental period affects several neurodevelopmental processes, ultimately impacting the overall functioning of the brain. Pro-inflammatory cytokines such as IL-1β and TNF-α impede neural cell migration, proliferation, and survival [69], resulting in a decrease in the populations of both neural and glial cells. This is supported by studies demonstrating that ethanol exposure during gestational days 11–21 disrupts neuronal survival and proliferation in the cortex [70]. In the hypothalamus, for example, ethanol-activated microglia prompted the interaction between microglia and POMC neurons, resulting in a significant loss of POMC neurons [71]. This effect occurs through the disruption of cell cycle regulation and impairment of the proliferation of neural progenitor cells [70].

In addition, neuroinflammation may severely compromise synaptic development and plasticity. Pro-inflammatory cytokines disrupt normal synaptogenesis and synaptic pruning by interfering with the CX3CL1–CX3CR1 signaling pathway [56]. Rat pups exposed to ethanol from postnatal days 2 to 9 exhibited compromised hippocampal synaptic plasticity, as evidenced by the diminished long-term potentiation and altered expression of synaptic proteins [72]. In one study, neonatal rats were exposed to ethanol from postnatal day 3 to 5, leading to distinct temporal inflammatory patterns in the cerebellum. These patterns were marked by elevated IL-1β levels during withdrawal periods (P4 and P6), with the highest increase in TNF-α being observed during the initial withdrawal period (P4) [65]. This correlates with the loss of Purkinje cells in the cerebellar vermis, a crucial component for coordinating motor function.

### 3.4. Long-Term Consequences of Ethanol-Induced Neuroinflammation

Neuroinflammation induced by ethanol persists after the initial exposure ends, leading to a chronic inflammatory state that can last throughout the development period. Studies have demonstrated that early exposure to ethanol leads to prolonged microglial activation and the increased production of pro-inflammatory cytokines, which persist into adolescence and adulthood [73,74]. The epigenetic programming of the immune system is the molecular basis for this persistence. A study demonstrated that neonatal rats exposed to ethanol from postnatal days 2 to 6 exhibited increased expression of pro-inflammatory molecules into adulthood [60]. This prolonged neuroinflammation occurs through epigenetic modifications, particularly histone H3K9 acetylation at the promoters of TNF-α and IL-6 in microglial cells [73]. These epigenetic changes create a primed immune state in the CNS, where microglia remain hypersensitive to subsequent inflammatory stimuli for a prolonged period after ethanol exposure stops. This persistent state of neuroinflammation affects the brain development processes, causing permanent structural and functional changes that persist throughout life.

The disruption of neural circuits and connectivity contributes to the characteristic behavioral and cognitive impairments observed in FASD [75]. Chronic inflammatory responses can cause extensive neurodegeneration, leading to irreversible changes in brain structure. These structural changes range among brain areas; the most visible effects are evident in the cerebellum, hippocampus, cerebral cortex, and white matter since these regions are more sensitive to damage produced by neuroinflammation following ethanol exposure. For example, ethanol exposure during early brain development significantly affects the cerebellum, where neuroinflammation is the leading player. A study has shown that ethanol exposure caused significant Purkinje cell loss in the cerebellar vermis, with regional differences in vulnerability [65]. The study indicated that postnatal ethanol exposure from day 3 to day 5 increased pro-inflammatory cytokines, primarily IL-1β, during the withdrawal phase. It also activated microglia, as evidenced by the transition to an amoeboid shape and elevated astrocytic GFAP expression. This neuroinflammation response coincided with Purkinje cell degeneration and measurable impairments in gait metrics that persisted into adolescence (PD45). This finding underscores that early ethanol exposure can cause enduring damage to brain areas, such as the cerebellum, potentially affecting essential brain functions.

The hippocampus is another brain region affected by chronic inflammatory responses. A study demonstrated the effect of maternal ethanol exposure on neonatal female rats, which experienced a significant elevation in inflammatory molecules in the hippocampus at postnatal day 8 compared to controls [76]. This study also observed lower corticosterone-binding globulin levels in neonatal rats exposed to ethanol, suggesting a reduced corticosterone reservoir associated with increased susceptibility to inflammation during this critical developmental period. This neuroimmune disturbance aligns with anatomical alterations following early ethanol exposure that caused noticeable changes in the cells, primarily a decrease in the number of pyramidal and granule neurons and the disruption of their functional capacities [72,77,78]. Reducing these vital neuronal populations could cause lasting changes in the hippocampal circuit’s formation and function. Persistent inflammatory mechanisms may mediate these structural changes, as the chronic activation of neuroinflammatory pathways disrupts normal glial cell function and compromises neuronal survival [79].

Early ethanol exposure also impacts the cerebral cortex, leading to permanent structural damage. Postnatal ethanol exposure causes significant cellular damage to the cerebral cortex, as evidenced by the large number of dead neurons, reduced neural network construction, and compromised neuronal growth [80,81,82]. These changes are particularly pronounced in prefrontal regions, which continue developing into adolescence and thus have extended vulnerability windows [83]. The resulting alterations in cortical architecture and connectivity permanently affect higher cognitive functions that rely on intact cortical circuits [84].

Furthermore, this persistent neuroinflammatory state impacts white matter development and myelination. A study found that exposure to ethanol during pregnancy induces neuroinflammation in offspring, marked by the increased expression of pro-inflammatory markers such as TLR4, NF-κB/p65, NLRP3 inflammasome, caspase-1, and IL-1β [79]. These inflammatory mediators reduce the levels of myelin proteins, including MAG and PLP, in the prefrontal cortex and hippocampus of mice prenatally exposed to ethanol, leading to lasting myelination loss that impairs cognitive function and neuronal transmission [79]. These white matter abnormalities are particularly pronounced in the corpus callosum, as demonstrated by the elevated radial diffusivity values in diffusion tensor imaging investigations [85]. The resulting hypomyelination irreversibly impairs the speed and efficiency of neural transmission between brain regions, disrupting the coordinated activity essential for complex cognitive and behavioral functions.

#### 3.4.1. Behavior Impairment in FASD

Cognitive functions are considered one of the primary characteristics associated with ethanol-induced neuroinflammation, which affects areas that depend on hippocampal integrity. Research utilizing mouse models has demonstrated that exposure to ethanol during development affects memory and the learning of spatial skills [15,86]. Rats exposed to ethanol showed a gradual decline in reference memory and spatial functioning as task difficulty increased [87]. Similarly, ethanol-treated mice exhibited deficiencies in delay-non-match-to-place spatial memory tests, which are tasks dependent on hippocampal learning [88]. The relationship between these cognitive impairments and neuroinflammations is supported by evidence that maternal binge-like ethanol exposure causes prolonged dysregulation of the neuroimmune response in offspring, potentially leading to substantial long-term impacts on memory and learning [79].

Further, motor function is also disrupted by ethanol-induced neuroinflammation. In human FASD patients, impairment is observed in postural balance and fine motor control tasks [89,90]. The findings are similar in rodent models, where mice exposed to ethanol during development demonstrated motor coordination deficits when tested on the accelerating rotarod in late adolescence. In addition to the cerebellum, which may be affected by neuroinflammation, as discussed above, dysfunction in the brain networks associated with motor skill learning may also occur [91,92], particularly hypomyelination in the corpus callosum, as indicated by increased radial diffusivity in imaging studies [85].

#### 3.4.2. Therapeutic Implications and Causal Evidence

The direct relationship between FASD-related impairment and neuroinflammation can be identified through studies that demonstrate how anti-inflammatory interventions can ameliorate or reverse cognitive and behavioral deficits. One of these interventions is utilizing minocycline antibiotic, which has potent anti-inflammatory properties, and provides evidence of a causal role in neuroinflammation in FASD. Treatment with minocycline reduced pro-inflammatory cytokines, such as MCP-1 and IL-6, and inhibited ethanol-activated microglia in a rodent model. The reduction in neuroinflammation promoted improved neurological outcomes by protecting neurons from ethanol-induced neuronal death in a rodent model [93]. Minocycline highlights the direct link between the inflammatory process and structural damage, as evidenced by its ability to reduce inflammation and prevent neural loss, which is seen in individuals with FASD.

Additional evidence of this association comes from cannabidiol (CBD) treatment during the peri-adolescent phase in a rodent model [94]. Administered CBD normalized ethanol-induced elevations in TNF-α and IL-6 levels in the hippocampus. Notably, this reduction in neuroinflammation significantly improved cognitive impairments in memory and executive functioning, indicating behavioral recovery [94]. Interestingly, CBD treatment enhanced cognitive performance during the peri-adolescence period, which is the timeframe following initial exposure, which suggests that targeting persistent inflammation might improve behavioral deficits.

Similarly, ibuprofen treatment in neonatal rats (PD4-9) reduced the ethanol-enhanced production of TNF-α and IL-1β by inhibiting cyclooxygenase-2 (COX-2), an enzyme that facilitates pro-inflammatory cytokine and chemokine release, in the dorsal hippocampus [95]. Reducing these inflammatory molecules improved trace fear memory performance, offering direct evidence that early neuroinflammation plays a role in cognitive impairments linked with FASD. These findings collectively suggest that inhibiting early neuroinflammatory responses may prevent or alleviate the behavioral and cognitive impairments associated with FASD, underscoring neuroinflammation as the primary mechanism underlying FASD pathology.

## 4. Gut Dysbiosis May Contribute to FASD Through Neuroinflammation

**Figure 1:**
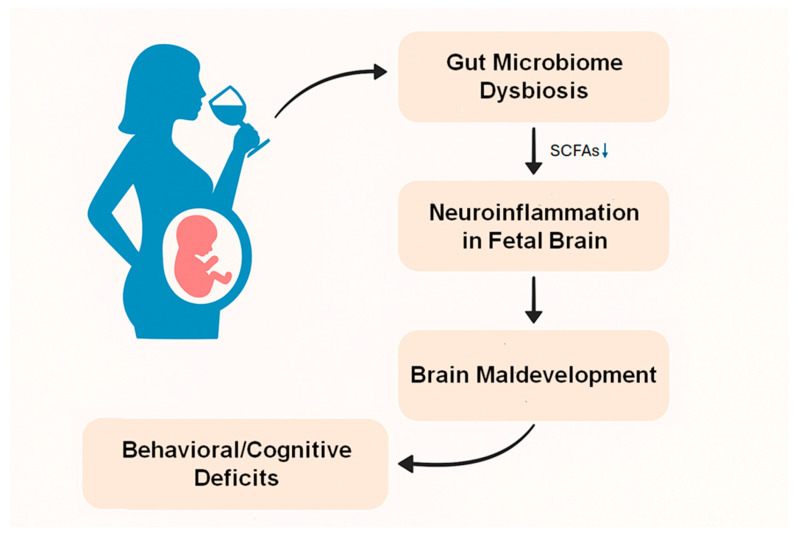
Maternal alcohol consumption induces gut microbiome dysbiosis and disturbs SCFA production, which may cause neuroinflammation in the fetal brain through the gut–brain axis, resulting in brain maldevelopment, and behavioral and cognitive deficits in FASD.

Gut microbiome dysbiosis resulting from ethanol exposure during pregnancy may contribute to the development of FASD (Figure 1). Maternal ethanol exposure triggers a complex pathophysiological cascade linking gut microbiome disruption to the onset of FASD through neuroinflammation. Ethanol simultaneously impacts two vital developmental systems during pregnancy. These two developmental systems are the fetal gut microbiome and the central nervous system. Recent studies in rat models indicate that ethanol consumption significantly alters the composition of the gut microbiome, particularly affecting bacterial families such as *Ruminococcaceae* and *Lachnospiraceae*, which are involved in the production of SCFAs [53]. These changes might have a significant impact as these bacteria produce butyrate, which has substantial neuroprotective and anti-inflammatory properties [53]. The timing of these changes is incredibly vital, as both the gut microbiome and the brain undergo concurrent developmental trajectories during gestation. They possess sensitive phases during which ethanol-induced disruptions in one system can profoundly influence the other through the gut–brain axis.

The interplay between gut dysbiosis and neuroinflammation in FASD establishes a self-sustaining cycle that may explain the ongoing neurodevelopmental deficits. Research using germ-free and antibiotic-treated murine models has demonstrated that SCFAs are crucial for the maturation and functionality of microglia [96]. When the production of these metabolites is disrupted, microglia display significant defects, including altered cell proportions and impaired innate immune responses [96]. The effects of SCFAs go beyond microglial function to encompass the maintenance of BBB integrity and the regulation of tight junction proteins [97]. The altered immune responses create an environment that sustains neuroinflammation beyond the initial ethanol exposure [98], potentially clarifying the long-term cognitive and behavioral effects of FASD.

Recent research from FASD and other neurodevelopmental disorders supports the therapeutic significance of the gut–brain axis. Studies using germ-free mice have demonstrated that disrupting the gut microbiome balance leads to increased BBB permeability and reduces the synthesis of tight junction proteins. However, these issues can be alleviated by restoring gut microbiota or introducing SCFA supplements [99]. Human clinical studies of microbiome transfer in individuals with ASD, a condition that shares common features with FASD, have gained some success, leading to enhanced behavioral and gastrointestinal symptoms among those affected with ASD, which can last up to two years after intervention [100]. Translating these interventions to human FASD populations presents challenges, especially regarding optimal timing and individual variation.

In addition to alterations in SCFAs, ethanol-induced gut dysbiosis also changes other metabolic pathways that are involved in amino acid production, carbohydrate metabolism, and vitamin synthesis [53]. For example, gut dysbiosis disrupts the synthesis of two main vitamins: folate and cobalamin. These vitamins are vital for brain development and DNA activities; however, their production is reduced as a result of early ethanol exposure [53]. Together with SCFAs, changes in these metabolites may lead to persistent neuroinflammation and enduring neurodevelopmental impairments.

Further, ethanol exposure during pregnancy impacts cellular mechanisms in the gut–brain inflammatory axis. Tuft cells are specialized intestinal cells that serve an immune sentinel function, recognizing pathogens through receptors like Sucnr1 and maintaining microbial homeostasis in cooperation with Paneth cells [101]. However, the tuft cell-Paneth cell circuit can be disrupted by dysbiosis resulting from ethanol exposure during pregnancy, consequently impairing the regulation of bacterial populations and contributing to inflammatory responses. This disruption promotes pathogenic bacterial overgrowth, with recent studies showing that ethanol exposure during pregnancy increased *Desulfovibrionaceae* populations [53]. The overgrowth of these bacterial families is correlated with impairments in the intestinal barrier and leads to inflammation [102]. They reduce sulfate to produce toxic hydrogen sulfide, which impacts disulfide bonds in the intestinal epithelium [102]. In addition, gut microbiome dysbiosis weakens the immune signaling and protective metabolites that normally protect the lung, increasing respiratory infection susceptibility [103]. This effect might create a secondary inflammatory pathway through the gut–lung axis bidirectional relationship, ultimately contributing to the etiology of FASD.

Moreover, human clinical studies have demonstrated that alcohol exposure during pregnancy triggers maternal placental inflammation in women. A recent human study demonstrated increased proportions of fetal placental villi macrophages (Hofbauer cells) and differential expression of 35 inflammatory genes in alcohol-exposed human placentas [104]. Additionally, clinical research has shown that maternal alcohol consumption during pregnancy significantly caused alterations in toll-like receptor (TLR) signaling in human offspring, with studies showing increased pro-inflammatory interleukin-1-beta production following TLR2 stimulation in peripheral blood mononuclear cells from infants exposed to alcohol [105]. These immune alterations in humans suggest that alcohol exposure during pregnancy primes both placental and systemic inflammatory responses, leading to altered corticotropin-releasing hormone (CRH) levels and maternal immune activation, ultimately exacerbating neuroinflammation [106].

## 5. Gut Microbiome Modulation Approaches: Mitigating Ethanol-Induced Neuroinflammation

Numerous studies have shown that improvements in gut microbiome significantly mitigate the detrimental effects of ethanol on animal models, underscoring a potential link between gut microbiome dysbiosis and neuroinflammation. Administering Lactobacillus rhamnosus GG (LGG) probiotics to a murine model diminished the systemic inflammation and cognitive deficits linked to chronic ethanol exposure [22]. Mechanistically, LGG restores gut microbiota’s ethanol-altered composition, reducing pro-inflammatory cytokines (IL-6, IL-1β, and TNF-α) in the intestine, bloodstream, and cerebral regions. Simultaneously, it enhances the production of neuroplasticity proteins crucial for cognitive function [22]. Considering these promising results, certain limitations need to be taken into consideration. One of these considerations is that probiotics show significant variability among individuals in terms of the effectiveness and persistence of colonization, influenced by host characteristics such as initial microbiome composition, which affects therapy outcomes [107]. Additionally, the effects of probiotics are often transient, necessitating continuous administration to sustain their beneficial effects [108].

Dietary supplementation with the SCFA butyrate has also enhanced ethanol-induced neuroinflammation and cognitive impairment in mouse models [23]. Butyrate functions as an HDAC inhibitor, promoting beneficial epigenetic modifications that enhance neuroprotection while suppressing microglial activation and pro-inflammatory cytokine production [43]. Also, butyrate helps restore gut barrier integrity and modulates the gut–brain axis disrupted by ethanol consumption [23]. Nevertheless, butyrate intervention therapy might have some limitations, including the dose-dependent increase in colonic visceral sensitivity observed in mouse models and the potential toxicity associated with developing intestinal tissue, where excessive SCFA accumulation may damage the intestinal mucosa in a maturation-dependent manner [109]. In addition, other side effects, such as nausea and its unpleasant smell, present challenges for therapeutic applications that should also be taken into account [110].

Furthermore, fecal microbiome transplantation (FMT, a procedure to transfer fecal microbiome from a healthy donor to a recipient) offers a promising approach to addressing ethanol-related issues. A study demonstrated its effectiveness in mitigating ethanol-induced depression, anxiety, and neuroinflammation [111]. It functions by restoring the gut microbiome composition and maintaining intestinal barrier integrity through the upregulation of tight junction proteins (ZO-1 and occludin) while reducing systemic inflammation, as evidenced by decreased lipopolysaccharide (LPS) levels and pro-inflammatory cytokines (TNF-α, IL-4, IL-18) [111]. FMT also increases serotonin (5-HT) levels in both intestinal and brain tissues and enhances the expression of 5-HT receptors (5-HT1A and 5-HT2A), thereby improving neurotransmission disrupted by ethanol exposure [111]. Despite these promising findings, its clinical implementation faces significant challenges. One of these considerations is safety, as there are differing adverse event profiles based on the route of administration (upper gastrointestinal routes: 43.6% adverse events; lower gastrointestinal routes: 17.7%). Serious complications were documented in 9.2% of recipients; in addition, there are no established standards for donor screening, inadequate long-term safety data, and the potential risks of its permanently altering the gut microbial composition [112].

## 6. Limitations and Considerations for the Clinical Translation of Microbiome-Based Interventions

Translating microbiome-based interventions from rodent models to human FASD populations presents significant challenges. There are substantial differences in bacterial species, with only 2.58% of bacterial species present in the gastrointestinal tract microbiome of both humans and mice [113]. In addition, there are significant differences in the timing of brain development between humans and rodents. While humans complete all three stages of brain development before birth, rodents experience their third stage of brain development 10 days after birth [114]. Furthermore, several factors can affect the composition of the human gut microbiome during pregnancy. These factors include diet, genetics, geography, the mode of delivery, and exposure to environmental elements and antibiotics [115]. In contrast, for rodent models these factors are controlled in laboratory settings. Therefore, translational barriers indicate that the findings from rodent models require cautious interpretation. They also underscore the necessity of human-specific research to validate the intervention approach, optimize the dosing protocol, and account for environmental factors that influence the human gut microbiome’s response to alcohol exposure and the therapeutic outcomes of intervention.

Determining an appropriate developmental stage for microbiome-based interventions is considered quite challenging. Preclinical and human studies suggest significant developmental periods when microbiome-based therapies may exhibit optimal efficacy. Heijtz et al. showed that early microbial colonization significantly impacts neurodevelopment and behavior [30]. Laue et al. emphasized that the first three years of life are pivotal for microbiome and brain development [32]. While numerous studies have shown that gut microbiome therapies can alleviate specific ethanol-induced effects, a significant gap still exists in knowledge regarding the most effective time of these interventions to prevent neurodevelopmental abnormalities in FASD. Specifically, studies comparing the efficacy of therapies at various developmental stages are insufficient; therefore, whether early postnatal interventions are more beneficial than those provided later is still unknown. The knowledge gap highlights the necessity for studies that investigate the developmental periods during which microbe-targeted interventions might be most effective in preventing or mitigating the neurological effects of maternal alcohol exposure.

## 7. Knowledge Gaps and Future Directions

Numerous critical knowledge gaps must be addressed regarding the involvement of the gut microbiome in FASD pathophysiology and the potential treatment approaches. A recent study reveals that maternal ethanol exposure considerably impacts the composition and diversity of the gut microbiome [49,53]. The mechanisms linking these microbial alterations to neurodevelopmental consequences are not well understood. Secondly, our understanding of the effects of the timing and duration of ethanol exposure during pregnancy on the offspring’s gut microbiome is limited. The significant sex-specific effects of microbiome changes in FASD remain mostly unexplored despite the recognized sex disparities in FASD outcomes. No studies have investigated the persistent changes in the gut microbiome that occur from early childhood to adulthood.

Future research should focus on establishing causal links between gut dysbiosis and FASD characteristics. Longitudinal studies require tracking of the gut microbiome composition and neuroinflammatory markers in offspring exposed to maternal ethanol to determine the temporal correlations. Intervention studies examining probiotics, prebiotics, or postbiotics during critical stages of development could assess their efficacy in reducing neuroinflammation and related behavioral issues and impairments. These studies must utilize comprehensive behavioral evaluations to connect microbiome-gut–brain alterations to functional outcomes. Finally, metabolomic investigations of microbial metabolites in both circulation and the central nervous system would facilitate the identification of specific mediators of neuroinflammation, potentially uncovering novel therapeutic targets for the treatment or prevention of FASD.

## 8. Conclusions

FASD is regarded as a significant public health concern, with persistent consequences for neurodevelopment and behavior. Recent studies demonstrate that ethanol exposure during pregnancy leads to persistent alterations in the gut microbiome, which may affect neuroinflammation and neurodevelopmental outcomes. Substantial changes in microbial composition, notably the decline in advantageous butyrate-producing bacteria and the increase in detrimental bacteria, indicate that the microbiome may significantly influence neuroinflammation and the pathophysiology of FASD. Preliminary evidence suggests that microbiome-based interventions may provide promising treatment alternatives; however, further studies are required to elucidate the mechanisms linking changes in the gut microbiome to FASD pathophysiology and to determine effective, personalized interventions. Future research should elucidate these mechanisms and develop evidence-based therapeutic strategies that account for the timing of the intervention and individual diversity in the intervention response.
